# Microbial impact on cholesterol and bile acid metabolism: current status and future prospects

**DOI:** 10.1194/jlr.R088989

**Published:** 2018-11-28

**Authors:** Aicha Kriaa, Mélanie Bourgin, Aline Potiron, Héla Mkaouar, Amin Jablaoui, Philippe Gérard, Emmanuelle Maguin, Moez Rhimi

**Affiliations:** UMR 1319 Micalis, INRA, Microbiota Interaction with Human and Animal Team (MIHA), AgroParisTech, Université Paris-Saclay, F-78350 Jouy-en-Josas, France

**Keywords:** gut microbiota, cholesterol metabolism, metabolic diseases, hypercholesterolemia

## Abstract

Recently, the gut microbiota has emerged as a crucial factor that influences cholesterol metabolism. Ever since, significant interest has been shown in investigating these host-microbiome interactions to uncover microbiome-mediated functions on cholesterol and bile acid (BA) metabolism. Indeed, changes in gut microbiota composition and, hence, its derived metabolites have been previously reported to subsequently impact the metabolic processes and have been linked to several diseases. In this context, associations between a disrupted gut microbiome, impaired BA metabolism, and cholesterol dysregulation have been highlighted. Extensive advances in metagenomic and metabolomic studies in this field have allowed us to further our understanding of the role of intestinal bacteria in metabolic health and disease. However, only a few have provided mechanistic insights into their impact on cholesterol metabolism. Identifying the myriad functions and interactions of these bacteria to maintain cholesterol homeostasis remain an important challenge in such a field of research. In this review, we discuss the impact of gut microbiota on cholesterol metabolism, its association with disease settings, and the potential of modulating gut microbiota as a promising therapeutic target to lower hypercholesterolemia.

Over the last few years, we have witnessed a myriad of original studies being dedicated to unraveling the role of gut microbiota in health and disease, such as inflammatory bowel diseases (1), obesity (2–4), type 2 diabetes (5), liver cirrhosis (6), and atherosclerosis (7–9). The human gastrointestinal tract (GIT) hosts a large number of distinct microbial communities, including bacteria, viruses, archaea, parasites, and fungi (10, 11). In a healthy state, interactions between these microorganisms and the host are largely symbiotic (12), as the gut microbiota shapes the development of the intestinal immune system (13) and influences the host metabolism [mainly through the production of bacterial metabolites as bile acids (BAs) and short-chain fatty acids] (14, 15). Conversely, changes in the gut microbiota composition/functions (known as dysbiosis) in combination with the classic genetic and environmental factors were shown to impact host health, thus triggering the development of several metabolic disorders, mainly CVDs (16). Recently, growing appreciation has been seen for a role of the gut microbiota in metabolic health, mainly in cholesterol homeostasis. The complex interplay between intestinal microbiota, their metabolites, and such diseases was also highlighted (17). Still, bacterially mediated pathophysiological mechanisms that impair cholesterol metabolism and other related metabolic traits remained, until now, poorly investigated (18). Surprisingly, most of the contemporary literature linking gut microbiota to host lipid metabolism and metabolic disorders was aimed at drawing causal inferences and defining microbial signatures for the disease. A better understanding of the mutual interactions regulating cholesterol metabolism will broaden the path to discover new targets for disease treatment. In this review, we shed more light on the impact of gut microbiota on cholesterol and BA metabolism in health and disease with a focus on the different microbial pathways involved and their functional basis.

## OVERVIEW OF CHOLESTEROL METABOLISM

**Fig. 1.**
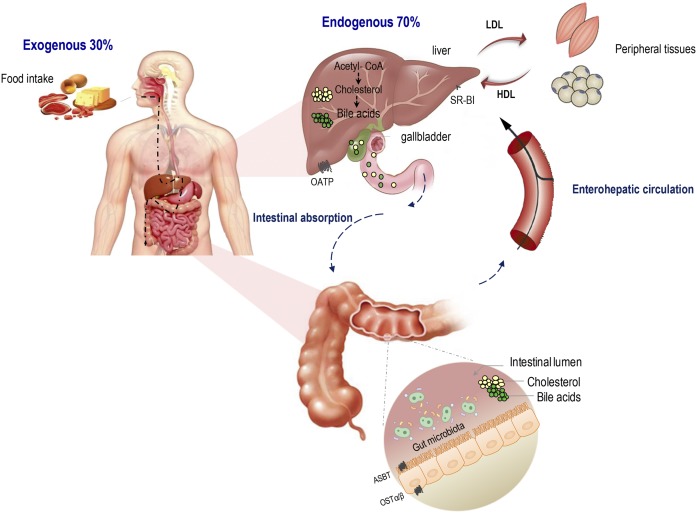
Cholesterol origins and metabolism. Dietary cholesterol or “exogenous” cholesterol accounts for approximately one-third of pool body cholesterol, the remaining 70% is synthesized exclusively in the liver through a series of multiple biochemical steps. Cholesterol is first converted into BAs, which are absorbed by the apical sodium-dependent BA transporter (ASBT) into enterocytes and secreted into the portal circulation via the basolateral BA transporter, organic solute transporter subunit α (OSTα). In the liver, cholesterol is converted into lipoproteins. Hepatic cholesterol enters the circulation as VLDLs, which are further metabolized to LDLs. LDL supplies cholesterol to peripheral tissues for metabolic purposes. HDL, on the other hand, transports cholesterol back to the liver either directly by interacting with hepatic SR-B1 or indirectly by trans­ferring the cholesterol to VLDL or LDL.

Beyond being an essential molecule for eukaryotic life as a structural building block for all cell membranes (19, 20), cholesterol is believed to serve as a genuine modulator of cell signaling (21) and neuronal conduction (22). It is also an essential precursor of several biomolecules, including steroid hormones, vitamin D, oxysterols, and BAs (23). Only about one-third of the body cholesterol is of dietary or “exogenous” origin (mainly animal products as eggs and red meat), the other two-thirds are synthesized within body cells and recognized as endogenous cholesterol (**Fig. 1**) (24). Virtually, all nucleated cells are able to synthesize their full complement of cholesterol; however, only the liver has the capacity to eliminate cholesterol via secretion into bile or conversion into BA (25). Together with the intestine, the liver controls the influx and the efflux of cholesterol in a coordinated manner, maintaining the whole-body cholesterol homeostasis. In the liver, part of free dietary as well as de novo-synthesized cholesterol is esterified to cholesteryl esters and packaged along with triglycerides and ApoB-100 into VLDLs to be secreted into the blood. The latter lipoproteins are further metabolized to form LDLs that are involved in the transport to peripheral tissues, as the case of VLDLs. Such lipoproteins containing ApoA-I mediate the reverse cholesterol transport from peripheral cells into the liver (26, 27). In fact, several reports described the key role of ApoA-I in the different steps of reverse cholesterol transport starting from the nascent HDL formation and their remodeling, via LCAT, to the HDL cholesterol delivery into the liver through scavenger receptor class B type 1 (SR-B1) (28, 29). Returning to the liver by HDL, cholesterol is further converted into BAs. Previously seen as simple fat emulsifiers, BAs are now known as critical modulators influencing a plethora of host processes, including lipid, glucose, and energy metabolism, through the activation of several nuclear receptors, namely, FXR, pregnane X receptor, vitamin D receptor, and one G protein-coupled receptor (TGR5) (30–33). Following a coordinated series of steps involving at least 17 different enzymes (34, 35), two primary BAs, chenodeoxycholic acid (CDCA) and cholic acid (CA), are synthesized from cholesterol in the liver. Prior to secretion, BAs are subsequently conjugated to either glycine or taurine, with a ratio of glycine to taurine of 3:1 in humans, and then stored in the gallbladder as mixed micelles along with cholesterol and phospholipids. Conjugation reduces BA pK_a_, making BAs more water soluble and much more able to fulfill their function as typical detergent molecules in the acid environment of the duodenum (36).

On consumption of a meal, the gallbladder contracts and releases BA micelles into the intestinal lumen to help solubilize cholesterol and fat-soluble vitamins. BAs are afterward extensively reclaimed by the distal ileum via the apical Na^+^-dependent transporter (ASBT), present on the enterocyte brush border. Intracellularly, intestinal bile-acid-binding protein (IBABP, FABP6) promotes BA transport to the basolateral membrane where BAs are effluxed by OST-α/β into the blood. Returning to the liver, BAs are taken up avidly by the Na^+^-taurocholate cotransporting polypeptide (NTCP) and, to a lesser extent, by organic anion transporters (OATPs) to be reconjugated and resecreted during the next course of digestion, thus completing a portal enterohepatic circulation (34) (Fig. 1). The combined effects of these coordinated steps mentioned above help to regulate not only the serum cholesterol level but also the whole-body cholesterol balance, which is maintained by fine interactions between cholesterol absorption, excretion, and synthesis (37).

## IMPACT OF GUT MICROBIOTA ON CHOLESTEROL METABOLISM

In addition to diet and the host’s genetic and environmental factors, bacteria present in the gut have recently been suggested to impact on cholesterol metabolism and play a key role in each pathway, ranging from cholesterol conversion into coprostanol to BA metabolism. Below, we give a concise overview of these bacterial pathways.

### Microbial conversion of cholesterol into coprostanol

**Fig. 2.**
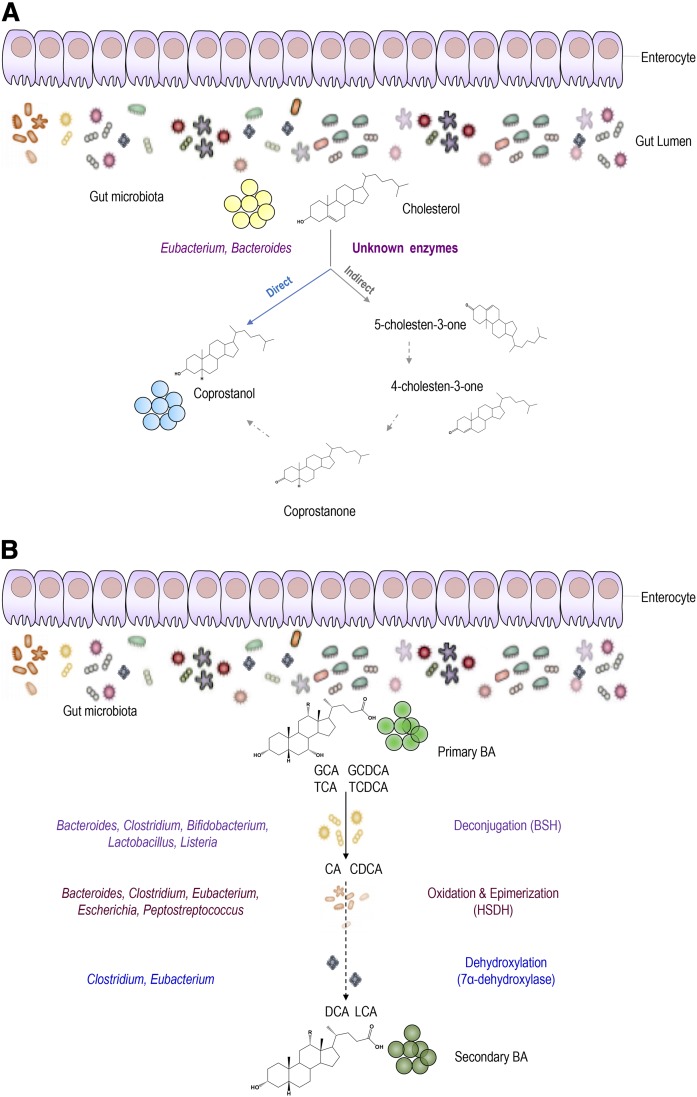
A: Bacterial conversion of cholesterol into coprostanol. Two major pathways are proposed for the conversion of cholesterol to coprostanol. The first pathway involves direct reduction of the 5,6-double bond. The second pathway starts with the oxidation of the 3β-hydroxy group and isomerization of the double bond to yield 4-cholesten-3-one, which undergoes two reductions to form coprostanone and then coprostanol. The main bacterial taxa carrying such a reaction involve *Eubacterium* and *Bacteroides*. However, bacterial enzymes are still unknown. B: Bacterial BA modifications in the host GIT. In the intestine, microbial enzymes from gut bacteria metabolize primary BAs into secondary BAs. Glyco-conjugated and tauro-conjugated CA and CDCA are first deconjugated via BSHs, epimerized, and then 7α-dehydroxylated to form secondary BAs (DCA and LCA). The main bacterial genera involved in BA metabolism include *Bacteroides*, *Clostridium*, *Bifidobacterium*, *Lactobacillus*, and *Listeria* in BA deconjugation; *Bacteroides*, *Clostridium*, *Eubacterium*, and *Peptostreptococcus* in the oxidation and epimerization of hydroxyl groups at C3, C7 and C12; *Clostridium* and *Eubacterium* in 7-dehydroxylation; and *Clostridium* and *Fusobacterium* in desulfation. GCA, glycocholic acid; TCA, taurocholic acid; GCDCA, glycochenodeoxycholic acid; TCDCA, taurochenodeoxycholic acid.

As much as 1 g of cholesterol originating from bile, diet, and desquamated cells enters the colon daily and is exposed to approximately 2 × 10^11^ to 5 × 10^11^ bacteria per gram wet weight of human feces (18). Cholesterol conversion to coprostanol by intestinal microorganisms was first reported in the 1930s (38) and shown to be established early in the first year of life in humans (39). The efficiency of microbial cholesterol-to-coprostanol conversion in human populations is bimodal with a majority of high converters (almost complete cholesterol conversion) and a minority of low or inefficient converters (coprostanol content representing less than one-third of the fecal neutral sterol content) (40, 41). Two major pathways have been proposed for this biotransformation (42). The first one is a direct stereospecific reduction of the 5,6-double bond of cholesterol, while the second one is an indirect transformation with at least three steps forming cholestenone and coprostanone as intermediates (**Fig. 2A**). Early attempts to isolate bacteria responsible for this conversion were unsuccessful (43), and only a few cholesterol-reducing microorganisms from rat cecum (44), hog sewage lagoon (45), and human feces were defined (43, 46). Most of the cholesterol-reducing bacteria isolated and characterized are members of the genus *Eubacterium*, except for *Bacteroides sp*. strain D8 (46) (Fig. 2A). Strains of *Bifidobacterium*, *Lactobacillus*, and *Peptostreptococcus* were also reported to reduce cholesterol to coprostanol (47, 48); unfortunately, they were not explored in vivo. Notably, cholesterol absorption takes place mainly in the upper small intestine, which harbors lactic acid bacteria (mostly *Lactobacillus*) able to significantly convert cholesterol into coprostanol (48). Lately, new bacterial phylotypes belonging to the *Lachnospiraceae* and *Ruminococcaceae* families have been associated with high coprostanol levels in healthy humans (49). Coprostanol production from the available cholesterol appears to be most efficient in the colon (50). Interestingly, Sekimoto et al. (50) described the existence of an inverse relationship between the blood cholesterol level and the coprostanol/cholesterol ratio in human feces, suggesting that produced coprostanol can modulate cholesterolemia. Moreover, several studies have reported that the rate of microbial cholesterol-to-coprostanol conversion in human populations is variable, as noted before, and is correlated with gut microbiota composition (41). In line with these observations, some bacteria are also linked to blood lipid levels (51). The poor absorption of coprostanol in the intestine is associated to its structure, which explains its very low uptake through the intestinal mucosa and its limited esterification in mucosal cells (52). Notably, a high efficiency of cholesterol to coprostanol metabolism was suggested to reduce the risk of CVDs (40, 53). Using mice lacking Niemann-Pick C1-like 1 (NPC1L1) treated with an LXR agonist, it was suggested that lipid level decrease was essentially linked to low cholesterol absorption and that production of coprostanol can protect the GIT against accumulated cholesterol (54). Moreover, it was demonstrated that mice lacking NPC1L1 display a different gut microbiota composition when compared with their wild-type littermates (55).

Unfortunately, the function, distribution, and abundance of bacteria carrying this conversion in the gut community are still unknown. Coprostanoligenic bacteria were suggested to use cholesterol as a terminal electron acceptor for energy, as originally proposed by Eyssen et al. (44), but the significance of such a conversion to the bacteria remains to be elucidated.

Given the inverse relationship between serum cholesterol levels and fecal coprostanol/cholesterol ratio (50) and the recent report highlighting a role for the gut microbiome in blood lipid levels (51), functional analysis of coprostanoligenic strains will be of great interest.

### Microbial entrapment of cholesterol

Cholesterol entrapment and incorporation into bacterial membranes was first noted in the early 1970s, as most of the mycoplasmas and strains tested were shown to require exogenous cholesterol for growth and incorporate large quantities of it into their cell membranes (56, 57). So far, the ability of bacterial uptake of cholesterol has been shown in vitro in several strains of *Lactobacillus* genera, including *Lactobacillus acidophilus*, *L. delbrueckii subsp. bulgaricus*, *L. casei*, and *L. gasseri* (58–61). Only distinct strains of both *Lactobacilli* and *Bifidobacterium* were suggested to perform such activity in vitro (62). Numerous studies were focused on the investigation of mechanisms of cholesterol entrapment by bacteria. In frame with this topic, it was proposed that cholesterol removal from the culture media is mainly associated to: *i*) binding to bacterial cell walls or its assimilation during growth; and *ii*) cholesterol precipitation (63, 64) (Fig. 2A). In addition to that, authors reported that most of the cholesterol taken up from the medium may have been incorporated into the bacterial cell membrane, as it remained intact inside the cells rather than being further metabolically degraded (64). Fascinating per se is the observation that even nongrowing and dead cells could remove cholesterol in vitro via binding of cholesterol to the cellular surface (62, 65, 66). Moreover, earlier studies have reported that some bacteria could produce exopolysaccharides that adhered to the cell surface and could absorb cholesterol (67). Kimoto-Nira et al. (66) previously suggested that the ability of cholesterol-binding is strain specific (65) and highly dependent on the chemical and structural properties of cell membranes (66). The incorporation of cholesterol into bacterial cell membranes increased the concentration of saturated and unsaturated fatty acids, leading to increased membrane strength and, subsequently, higher cellular resistance toward lysis (67).

Whether cholesterol entrapment may offer a protective effect to bacteria in the intestinal environment remains unclear, as the mechanism is still unknown. Additional studies are needed to define the influence of these strains on the intestinal microflora and the overall metabolic activity of the gut.

### Microbial metabolism of BAs

Bacterial metabolism of BAs represents one of the most intriguing relationships linking gut microbiota to the host. While most BAs are efficiently absorbed and recycled back to the liver, around 5% of the total BA pool serve as a substrate for bacterial metabolism in the GIT and constitute the major route for cholesterol excretion from the body (18). Below, we outline the main BA biotransformations by human intestinal bacteria.

### Deconjugation

On their side chain, BAs undergo deconjugation to form unconjugated BAs as well as free glycine or taurine residues (68). The enzymatic hydrolysis of the C-24 *N*-acyl amide bond, referred to as deconjugation, is catalyzed by bile salt hydrolase (BSH) enzymes in the small intestine, a process that continues to near completion in the large bowel (Fig. 2B) (69, 70). BSH activity has been widely detected in several bacterial genera, including *Clostridium*, *Bifidobacterium*, *Lactobacillus*, *Bacteroides*, *Enterococcus* (71, 72), and possibly many others. Deconjugation of bile salts increases their pK_a_ to ∼5, making them less soluble and less efficiently reabsorbed. This results in higher excretion of free BA into the feces, an amount that must be replenished by de novo synthesis from cholesterol (69). Although several hypotheses have been proposed (as follows), the precise benefits of this transformation to the bacterium are still a subject of controversy and appear to vary between bacterial isolates. Proposed benefits include the use of glycine from glycocholic acid as an energy, carbon, and/or nitrogen source and of taurine from taurodeoxycholate as a sulfur source (69). A role of taurine as a nitrogen source was also noted as the transcription of the *Bifidobacterium longum* bsh gene was coupled to the glutamine synthase adenyltransferase gene (glnE), which is part of the nitrogen regulation cascade (73). Deconjugation has also been proposed to play a role in bile detoxification, as it yields free BAs that may help to negate the pH-drop by recapturing and exporting the cotransported proton (74). It may also enhance gastrointestinal persistence in which the combined roles of BSH-positive bacteria and those entrapping cholesterol into their membranes may facilitate their survival in the GIT (67, 70).

### Oxidation and epimerization of 3-, 7-, and 12-hydroxyl groups

The oxidation and epimerization of the 3-, 7-, or 12-hydroxyl groups of BAs are carried out by the hydroxysteroid dehydrogenases (HSDHs) of intestinal bacteria. Epimerization of BA hydroxyl groups is a reversible change in stereochemistry from the α to the β configuration (or vice versa), involving the generation of stable oxo-BA intermediates (75, 76). This process can be performed by a single species of bacteria containing both α- and β-HSDHs (intraspecies) or by proto-cooperation between two species, one having an α-HSDH and the second containing the β-HSDH (interspecies). So far, HSDH activity has been confirmed in a diverse variety of bacteria, including *Bacteroides* (77), *Eubacterium* (78), *Clostridium* (79, 80), *Bifidobacterium* (81, 82), *Lactobacillus*, *Peptostreptococcus*, and *Escherichia* (80, 83). Notably, epimerization of BA hydroxyl groups was proposed to confer a protective effect for some bacterial species, as it reduces BA toxicity. Generated ursocholic acid (7β-hydroxy), for instance, is less hydrophobic than chenocholic acid (7α-hydroxy) and presumably less deleterious to cell membranes (84). Whether intestinal bacteria benefit from such reactions is still a matter of speculation.

### 7α-Dehydroxylation

In the colon, bacterial 7α-dehydroxylases convert primary BAs, CA (with hydroxy groups at C-3, C-7, C-12), and CDCA into deoxycholic acid (DCA) and lithocholic acid (LCA), respectively (69). Both are absorbed to some extent and returned to the liver (61). DCA is reconjugated and reabsorbed in the ileum, similarly to primary BA. In the form of its glycine and taurine conjugates, DCA was shown to account for more than 20% of the total biliary BAs in humans (68). LCA, on the other hand, never constituted more than 5%, as it is largely excreted in the feces once conjugated to sulfite in the liver (69). Quantitatively, 7α-dehydroxylation is the most important bacterial biotransformation, seeing that secondary BAs predominate in human feces (69, 85). Surprisingly, only distinct members of the *Eubacterium* and *Clostridium XIVa* cluster were shown to undergo this reaction (69, 71, 86, 87). Given the lack of redundancy in this ecosystem, any perturbations to these bacterial groups were suggested to likely influence host metabolism (88). Because 7α-dehydroxylation is a net reductive process, it was suggested to serve as a key electron-accepting reaction in the energy metabolism of dehydroxylating bacteria (69).

### Esterification and desulfation of BA in the gut

BA esters or saponifiable derivatives of BAs have been reported to account for 10–30% of the total fecal BA content in humans (89). However, little is known about the role of gut bacteria in carrying such a reaction. Bacterial genera responsible for BA desulfation include *Clostridium* (90, 91), *Peptococcus* (92), and *Fusobacterium* (93). To date, this reaction mechanism has not been unleashed, and the enzymes have not been characterized.

## DYSBIOSIS, CHOLESTEROL DYSREGULATION, AND METABOLIC DISEASES

Growing evidence suggests that gut microbiota may affect lipid metabolism and function as an environmental factor to influence the development of many metabolic diseases (6–9, 94, 95). Several mediators were hypothesized to link changes in gut bacteria to such diseases, including alterations in gut microbiota and subsequent changes in BA metabolism (as is the case for hepatic cirrhosis) and production of specific metabolites (atherosclerosis).

### Hepatic cirrhosis

Recent reports have shown potential mechanisms explaining how dysbiosis may affect BA metabolism and impact upon disease state (96–98). With advancing liver disease and cirrhosis, several taxonomic groups, including *Lachnospiraceae*, *Roseburia*, *Ruminococcaceae*, and *Blautia*, were reported to decrease in addition to the reduction in primary BA concentrations in the intestine due to the liver problems. As these taxa include members with BA 7α-dehydroxylation activity, secondary BA rates were also significantly lower in cirrhotic patients relative to healthy controls (98). Surprisingly, this decrease in the BA pool entering the intestine appears to promote the overgrowth of distinct pathogenic and pro-inflammatory members of *Enterobacteriaceae* and *Porphyromonadaceae* (97). Of note, a direct relationship was previously reported between such bacteria and cognitive impairment in cirrhotic patients (97). A recent comparison of gut microbial genome content between cirrhotic and healthy individuals suggests the enrichment for genes involved in ammonia production and manganese transport systems, each of which is suggested to play a mechanistic role in the cognitive problems associated with liver cirrhosis (6).

### Atherosclerosis

Several significant associations between distinct microbial taxa and atherosclerosis have been highlighted recently (17, 99, 100), thus strengthening the evidence for atherosclerosis as a microbiota-associated disease. Although pathogenic bacteria have been previously associated with such disease (9, 99, 100), the composition and functional alteration of commensal microbiota in relation to atherosclerosis were recently examined (17). Different pathways by which microbiota might affect atherogenesis were reported previously (9). These include local or distant infection of the host, alterations in cholesterol metabolism by gut microbiota, and the production of microbial metabolites. Microbial processing of specific dietary components (choline/carnitine) to trimethylamine (TMA), which is further metabolized to TMAO in the liver, has been previously associated with atherosclerosis in several studies (101–105) and is suggested to play a crucial role in the disease. In fact, TMAO upregulated several macrophage scavenger receptors (CD36 and SR-A1) associated with atherosclerosis. Furthermore, the use of germ-free (GF) mice proved the effect of diet and gut microbiota in the TMAO production linked to macrophage cholesterol accumulation and foam cell formation (101, 102). Dietary supplementation with the TMA-containing precursor (choline or carnitine) and dietary TMAO directly were each shown to enhance aortic root atherosclerotic plaque in mice (101). In humans, elevated plasma levels of TMAO were strongly associated with an increased risk of CVD (106–112). Indeed, numerous TMAO-associated proteins were identified as involved in the process of platelet aggregation (104, 110). Although not entirely clear, TMAO was suggested to modulate cholesterol and sterol metabolism at multiple sites in vivo (101, 102). In the liver, TMAO reduced the expression of key enzymes and multiple BA transporters (Cyp7a1, Oatp1, Oatp4, and Ntcp) and reduced the BA pool size (101).

Prior studies have suggested that multiple bacterial strains can metabolize carnitine and choline in vitro (113, 114), but specific commensal species that contribute to TMAO formation remain largely unknown. Recently, species of several bacterial taxa (*Prevotella* and *Bacteroides*) were shown to be associated with both plasma TMAO and dietary status (100, 102). A genus within the *Coriobacteriaceae* family (*Colinsella*) was also reported to be enriched in patients with symptomatic atherosclerosis (100). Of note, patients with atherosclerotic heart disease have higher cholesterol absorption and reduced fecal neutral steroid (115, 116), which according to the findings of Martinez et al. (117) was suggested to be linked to the increase of such genus.

Three main TMA-synthesis pathways have been described involving each distinct enzyme complex (CutC/D, CntA/B, and YeaW/X) (118–120). Recently, additional human bacterial taxa that exhibit choline TMA-lyase (CutC) and carnitine oxygenase (CntA) were further uncovered (121). Furthermore, microbial choline processing was proposed to confer certain advantages for bacteria in a complex environment (122, 123). Of interest, an association between atherosclerosis and gene abundance related to the TMA-synthesis metabolic pathway (mainly those encoding TMA lyases as YeaW/X) was lately described but further studies are certainly needed (17).

Whether via direct pharmacological inhibition of microbial enzymes involved in TMA production, dietary intervention, or modification of the microbial community with pro- or prebiotics, targeting the gut microbial TMAO pathway as a treatment strategy has the potential to decrease the risk of atherosclerosis. Further studies in animal models will thus be of importance to ensure causality, define precise mechanisms of action, and identify culprit bacteria in such disease.

## MICROBE-BASED STRATEGIES FOR CHOLESTEROL-LOWERING EFFECTS

### BSH-active bacteria as a cholesterol-lowering agent

Partly owing to their cholesterol-lowering effects and also being part of gut microbiota, BSH-active bacteria are now largely used as food supplements or “drugs” in this day and age. A growing number of people with hypercholesterolemia or CVDs have used these products containing so-called “friendly” bacteria. Lately, increased BSH activity was shown to disrupt micelle formation and absorption, thus resulting in a significant decrease of cholesterol level. Such a single widely distributed function of gut microbiota could not only significantly influence lipid metabolism but also weight gain and cholesterol levels in the host (70, 124, 125). As mentioned in Joyce et al. (124), high-level expression of cloned BSH enzymes in the GIT of conventionally raised mice resulted in a significant decrease in the host’s weight gain, plasma cholesterol, and liver triglycerides, demonstrating the overall impact of BSH activity on host physiology. In line with this data, a combination of probiotic strains, VSL#3 (*Lactobacilli*, *Bifidobacteria*, and *Streptococcus salivarius subsp. thermophilus*), was also found to improve lipid profiles in mice (126). This commercially available mixture of bacteria promotes BA deconjugation and fecal excretion, and increases hepatic BA synthesis via downregulation of the FXR/FGF15 axis (126) (**Table 1**). An extra piece of the puzzle in this captivating cross-talk was also revealed when Li et al. (30) reported that pharmacologically reducing the genus of *Lactobacillus* within the gut was linked to a decrease of the BSH activity, allowing the reduction of obesity induced by high-fat diet in mice. Such an effect was proposed to be mediated by the accumulation of intestinal tauro-β-muricholic acid that has been evidenced as a natural FXR antagonist (30). Recently, the colonization of gnotobiotic mice with *Bacteroides thetaiotaomicron* and its corresponding mutant for BSH activity demonstrated an alteration of BA metabolism, including higher cecal TβMCA levels and lower liver and plasma lipid levels (127). At a transcriptional level, the observed modifications in the BA pool were reported to modulate the expression of genes involved in both lipid uptake and glucose metabolism, as well as those related to circadian rhythm and immune response (127).

**TABLE 1.** Summary of the major studies investigating the relationship between probiotics and hypercholesterolemia

Probiotic Strain	Animals/Subjects	Dose and Time of Intervention	Effects	Other Parameters	Possible Mechanisms	References
*Lactobacillus lactis subsp. lactic* IS-10285	Rats	2.7 × 10^8^ CFU/ml daily for 12 days	Decreased TC and LDL-c	Increased excretion of BA in feces	BA deconjugation	(133)
*Lactobacillus plantarum* KCTC3928	Mice	10^9^ CFU/ml for 4 weeks	Decreased LDL cholesterol level	Increased fecal BA excretion	BA deconjugation	(134)
*Lactobacillus acidophilus* ATCC 4962, mannitol, FOS, inulin	Pigs	1 g/day for 8 weeks	Decreased plasma total cholesterol	—	Cholesterol binding to cell walls	(62)
*Eubacterium coprostanoligenes*	Rabbits	2 × 10^7^ CFU/ml	Decreased plasma total cholesterol	—	Conversion of cholesterol into coprostanol	(135)
*Lactobacillus rhamnosus* SKG34 and FBB42	Rats	10^8^ CFU/ml for 4 weeks	Decreased TC, HDL-c and TG	—	NR	(136)
*Lactobacillus reuteri* NCIMB 30242	114 subjects	5 × 10^9^ CFU taken twice per d over 6 weeks	Reduced TC, LDL-c and nonHDL-c	—	BA deconjugation	(130)
VSL#3	60 subjects	112.5 × 10^9^ CFU/capsule for 6 weeks	Reduced TC, TG, LDL and VLDL	Increased fecal excretion	BA deconjugation	(128)
*Enterococcus faecium* CRL 183 and *Lactobacillus helveticus 416*	49 subjects	10^8^ and 10^9^ CFU/ml for 42 days	Decreased TC, LDL-c, nonHDL-c and CT/HDL-c ratio	—	NR	(137)

TC, total cholesterol; LDL-c, LDL cholesterol; HDL-c, HDL cholesterol; TG, triglycerides; NR, not reported.

Using a double-blinded placebo-controlled randomized study involving 114 subjects with high cholesterol levels, the same author reported a decrease in total cholesterol and LDL-C level by nearly 9% and 5%, respectively, after consumption of yogurt containing BSH-active *Lactobacillus reuteri* NCIMB 30242 (128–130) (Table 1). It is of interest that this strain is the first commercial cholesterol-busting probiotic ready for the US food, beverage, and supplement markets.

Not surprisingly, several reports are nowadays devoted to screening potential probiotic bacteria for BSH activity as potential biotherapeutics for metabolic diseases. However, these studies focusing on the influence of a single factor for strain selection (deconjugation in this case) are insufficient to justify a cholesterol-lowering effect of these strains.

### Lactic bacteria as a hypocholesterolemic agent

Cholesterol entrapment has been reported for numerous strains and was suggested to result in decreased availability of cholesterol for absorption, thus leading to reduced serum cholesterol. In fact, consumption of *Bifidobacterium bifidum*, which was shown to assimilate cholesterol in vitro, reduced serum cholesterol in hypercholesterolemic human subjects (60) (Table 1). *Lactococcus lactis* KF147 and *Lactobacillus plantarum* Lp81 were also reported to reduce cholesterol level by 12% (131). But, seeing that both bacteria exhibit BSH activity, it was difficult to attribute this effect to bacterial entrapment of cholesterol. Unfortunately, most of the in vivo trials done so far are based on in vitro trials and focus only on verifying the hypocholesterolemic effects shown in vitro, rather than the mechanisms involved. Interestingly, it was reported that inhibition of NPC1L1 by ezetimibe using GF and specific pathogen-free mice allows a decrease of intestinal cholesterol absorption and a decrease of fecal cholesterol excretion in GF mice correlated with a reduction of blood and hepatic cholesterol levels. Therefore, targeting the gut microbiota has been proposed as a promising strategy to lower cholesterolemia, probably in combination with other hypocholesterolemic drugs (132). Table 1 summarizes the main in vivo trials with the mechanism proposed for each hypocholesterolemic effect.

## CONCLUSIONS AND FUTURE PROSPECTS

In conclusion, current evidence has given some credence to the impact of gut microbiota on cholesterol metabolism and its contributory role in the development of metabolic diseases. Actually, microbiome profiling and fecal transplantation have both proved the key role of gut microbiota in cholesterol management, which may be a risk factor for CVDs once dysregulated. The analysis of these bacterial actions on cholesterol has allowed the identification of microbial pathways of cholesterol metabolism. Still, this field of research remains poorly studied, as many pathways still need to be unleashed and the involved enzymes identified. The determination of the molecular basis of these mechanisms of action will promote the use of intestinal bacteria as a powerful cholesterol-lowering agent. Such an approach can be combined with the existing attractive strategies, including phytosterols, to increase their efficiencies. Accumulating data from populations all over the world will certainly promote the opportunity to investigate the role of a functional microbiome on the host’s well-being, which constitutes a challenge to develop specific hypocholesterolemic therapies.
